# The Impact of Sedentary Lifestyle on the Psychological Well-being of Hospital Administrative Staff

**DOI:** 10.1192/j.eurpsy.2025.2159

**Published:** 2025-08-26

**Authors:** K. Imen, R. Nakhli, L. S. Chaibi, M. Bouhoula, A. Chouchane, M. Maoua, H. Kalboussi, O. El Maalel, S. Chatti, A. Aloui, N. Mrizak

**Affiliations:** 1 Occupational Medicine Department, University of Sousse, Faculty of Medicine Ibn Al Jazzar, Sousse; 2 D Psychiatric Department, Razi Hospital, Tunis, Tunisia

## Abstract

**Introduction:**

Sedentary behavior, a leading cause of preventable mortality in developed nations, has been linked to a range of health problems, including cardiovascular disease and depression. While the benefits of leisure-time physical activity are well-established, the impact of sedentary behavior within the workplace, particularly in the healthcare sector, remains under-explored.

**Objectives:**

To study the relationship between sedentary behavior and mental health among.

**Methods:**

A cross-sectional study was conducted among administrative staff at Farhat Hached University Hospital, Sousse, from March 2024 to June 2024. Data were collected through an anonymous, self-administered questionnaire written in French and distributed to the administrative staff of Farhat Hached University Hospital. It included six sections covering sociodemographic characteristics, lifestyle habits, medical data, physical activity. Mental health, was assessed using the Hospital Anxiety and Depression Scale (HAD) and the Perceived Stress Scale (PSS).

**Results:**

A total of 85 questionnaires were completed by administrative staff at Farhat Hached University Hospital. The majority of participants were female (sex ratio of 0.42), with an average age of 47.1 ± 8.2 years. Higher education levels were represented by 66 participants, and only 24 engaged in professional physical activity (<30 minutes per day). The average seniority at the institution was 19.91 ± 9.2 years. Administrative managers (25.9%) and administrative staff (20%) were the main professional categories. A significant proportion of participants exhibited signs of anxiety (25.8%) and depression (32.9%). The average perceived stress score was 17.75 ± 4.95. A significant association was found between anxiety and low levels of physical activity at work (p<0.001). However, no significant association was observed between perceived stress and physical activity at work.

**Conclusions:**

Sedentary behavior at work is associated with a high prevalence of anxiety and depressive disorders among hospital administrative staff, highlighting the importance of promoting physical activity to improve mental health.

**Disclosure of Interest:**

None Declared

